# Regenerative Potential of Endometrial Stem Cells: A Mini Review

**Published:** 2015-01

**Authors:** Farnaz Ghobadi, Davood Mehrabani, Golnoush Mehrabani

**Affiliations:** Stem Cell and Transgenic Technology Research Center, Shiraz University of Medical Sciences, Shiraz, Iran

**Keywords:** Endometrial stem cells, Regenerative medicine, Aesthetic medicine

## Abstract

Recent findings in stem cell biology have opened a new window in regenerative medicine. The endometrium possesses mesenchymal stem cells (MSCs) called endometrial stem cells (EnSCs) having specific regenerative properties linked to adult stem cells. They contribute in tissue remodeling and engineering and were shown to have immuno-modulating effects. Many clinical trials were undertaken to ascertain the therapeutic potential of EnSCS. In this mini review, we showed that EnSCs are readily available sources of adult stem cells in the uterus that can be highlighted for their renewable multipotent and differentiation properties. This cell population may be a practical solution of choice in reproductive biology, regenerative medicine and autologous stem cell therapy.

## INTRODUCTION

Adult stem cells are undifferentiated cells observed in several adult tissues. They have self-renewal and differentiation properties into one or more lineages, possess a high proliferative potential,^1^ have clonogenicity or colony forming unit (CFU) activity, and participate in tissue reconstitution during aging and for damaged tissues.^2^^,^^3^ Adult stem cells maintain tissue homeostasis by provision of replacement cells in routine cellular turnover and for repair of damaged tissues.^4^ Embryonic stem cells (ESCs) as the other most important division of stem cells have also great proliferation ability and controlled differentiation properties.^5^ But the possibility for immune rejection^6^ and the fear for appearance of teratomas^7^ for these cells caused a major obstacle for their clinical application. 

Adult stem cells have been isolated from tissues such as adipose tissue,^8^ umbilical cord blood,^9^ placenta,^10^ dermis,^11^ cardiac muscle,^12^ corneal limbus,^13^ periodontal ligament,^14^ dental pulp^15^ and endometrium.^16^ The endometrium harbors epithelial and fibroblast-like stromal cells, and is physiologically divided into the functionalis and basalis layers.^17^ Endometrium and myometrium are the two histological divisions of uterus in most mammalian species as essential tissue in reproduction.^18^ Mammalian endometrium is a dynamic tissue that during the reproductive life has cyclical periods of regeneration and regression.^19^ There is a monthly preparation of the tissue to receive the fertilized egg which is associated with a period of hyperproliferation and angiogenesis.^20^ The lining in the tissue expands by 5-7 mm in thickness within each menstrual cycle.^21^ In rodents, the epithelium undergoes identical cycles of proliferation in response to ovarian hormones.^18^

Studies on adult stem cell biology in uterine tissue lag far behind other areas of stem cell research despite the fact that, the uterus undergoes the most extensive proliferative changes and remodeling in adult mammals.^18^ In this tissue, the presence of stem cell populations called endometrial stem cells (EnSCs) was shown to participate in regenerative activities^22^ identical to tissues such as adipose, bone marrow, intestine and skin where mesenchymal stem cells (MSCs) have already been identified with regenerative potentials.^23^^-^^26^

EnSCs are in quiescent state which was confirmed by nanoparticle labeling studies.^27^^,^^28^ EnSCs are derived from endometrial biopsies and were shown to display properties such as clonogenicity, long-term culturing capability, multilineage differentiation potential,^29^^,^^30^ expression of CD146, CD90, CD73, CD105, MSI1, NOTCH1, and SOX2; and the lack of CD34 and CD14 expression.^31^ EnSCs are MSCs capable of differentiation properties of mesodermal and ectodermal lineages^32^ such as hepatocytes,^33^ neural cells,^34^^-^^39^, osteoblasts,^40^^-^^44^, smooth muscle,^45^ cartilage,^46^ heart muscles,^47^ adipocyte,^48^^,^^49^ megakaryocytes,^50^ and pancreatic tissues^51^ providing the potential for their clinical application.^52^^,^^53^

The isolation and culturing of these cells in mouse,^54^ guinea pig,^55^ primates,^56^ and cattle^57^ were previously reported. In post-menopausal women, EnSCs revealed comparable properties to premenopausal EnSCs regarding self-renewal in vitro too.^24^^,^^58^^,^^59^ The eutopic and ectopic characteristics of EnSCs were compared and was shown that ectopic EnSCs displayed a higher ability of cell migration, invasion and formation of new blood vessels.^60^ Not all of the reparative potential of EnSCs are related to their proliferation and differentiation features, but also their other properties such as immunomodulatory capability^61^ can make them proper candidates in the treatment of some autoimmune associated degenerative diseases like MS or CNS inflammation.^62^ EnSCs were shown to have the potential to be ‘off the shelf’ clinical reagents for the treatment of heart failure.^63^^,^^64^

The immunosuppressive mechanisms by which EnSCs reduce neuroinflammation was shown through the impairment of Th17 and Th1 T CD4 cells.^65^ The ability of EnSCs to differentiate into dopamine-producing neurons was demonstarted while *in vitro* cultures exhibited neuronal morphology with electrophysiological features resembling the dopamine-producing neurons and expressing markers of neural cell phenotype.^66^ Differentiation of these cells into efficient cholinergic neurons was noticed in presence of bFGF and NGF.^67^ The expression of neuronal markers such as MAP2, β3-tub and NF-L proteins in EnSCs cultured for 28 days at the presence of bFGF, PDGF and EGF signaling molecules was previously reported.^68^

EnSCs were shown to provide a therapeutic benefit in the primate model of Parkinson’s disease.^69^ The autologous implantation of EnSCs can lead to endometrial regeneration and restoration of menstruation and they can be a promising novel cell based therapy for refractory Asherman’s syndrome.^70^ In obese women with reproductive failure, the deficiency in clonogenic EnSCs denote to the important role of these adult stem cells.^71^

These cells were transplanted into the peri-infarct zone while resulted into a decrease in apoptosis and an increase in cell proliferation through activation of AKT, ERK1/2 and STAT3 and inhibition of p38 signaling denoting to regenerative role of EnSCs in the tissue.^72^ One of the promising regenerative capacity of EnSCs is their role in reconstruction of soft tissue defects.^73^ Ai et al. showed that human EnSCs treated with adipogenic media revealed their potential in regenerative therapies while these cells expressed PPARa at mRNA level.^74^ When EnSCs were inserted in a gelatine/apatite nanocomposite biomimetic scaffold in cranial bone defects of mice, there was a potential for these cells as regenerative tools in repair of hard tissues.^75^ In pelvic organ prolapses when mesh scaffolds were seeded with EnSCs, a significantly more neovascularization and less macrophages in the affected area were visible.^76^

Isolation of multipotent EnSCs from menstrual blood called menstrual blood mesenchymal stem cells (MBSCs) has also been recently reported.^56^ As EnSCs were used in tissue engineering and many clinical trials to ascertain their therapeutic potential, these cells are considered a readily available source of adult stem cells in the uterus that can be highlighted for their renewable multipotent and differentiation properties. So this cell population can be considered as a practical solution of choice in reproductive biology, regenerative medicine and autologous stem cell therapy.

## CONFLICT OF INTEREST

The authors declare no conflict of interest. 

## Note

Please cite this paper as:

Ghobadi F, Mehrabani D, Mehrabani G. Regenerative Potential of Endometrial Stem Cells: A Mini Review. World J Plast Surg 2015;4(1):3-8.
